# Fracture of the Modular Neck in Total Hip Arthroplasty

**DOI:** 10.1155/2015/591509

**Published:** 2015-07-22

**Authors:** A. Hernandez, A. Gargallo-Margarit, V. Barro, I. Gallardo-Calero, A. Sallent

**Affiliations:** Orthopaedic Surgery Department, Vall d'Hebron Hospital, 08035 Barcelona, Spain

## Abstract

Modularity of the components in total hip arthroplasty has had an increase in popularity in the last decades. We present the case of a 53-year-old man with a history of avascular necrosis of the femoral head due to a hypophyseal adenoma. A total hip modular arthroplasty was implanted. Three and a half years after the surgery the patient attended the emergency room due to acute left hip pain with no prior traumatism. Radiological examination confirmed a fracture of the modular neck. A revision surgery was performed finding an important pseudotumoral well-organized periprosthetic tissue reaction. Through an extended trochanteric osteotomy the femoral component was removed, and a straight-stem revision prosthesis implanted. There are several potential advantages when using modularity in total hip arthroplasty that surgeons may benefit from, but complications have arisen and must be addressed. Various circumstances such as large femoral head with a long varus neck, corrosion, patient's BMI, and activity level may participate in creating the necessary environment for fatigue failure of the implant.

## 1. Introduction

Modularity of the components in total hip arthroplasty has had an increase in popularity in the last decades [1, 2]. Its success has been mainly due to the versatility it offers. Surgeons have benefited from the potential advantages including altering leg length, offset, and anteversion as well as optimizing the biomechanics thus reducing wear, impingement, and luxation risk [3, 4]. Nonetheless, complications have been reported in the literature, which confer modularity some disadvantages that ought to be taken into consideration. Fretting, crevice and galvanic corrosion, component loosening, and fracture have all been associated with modularity. The use of large diameter metal on metal bearing has also been reported as a source of corrosion and a source of metal debris [5, 6]. Mid-term results in modularity have reported some complications and the issue is not without controversy [7–10]. Recently, there have been a few cases reported on fracture at the modular neck-stem taper junction [11, 12].

## 2. Case Report

**Figure 1:**
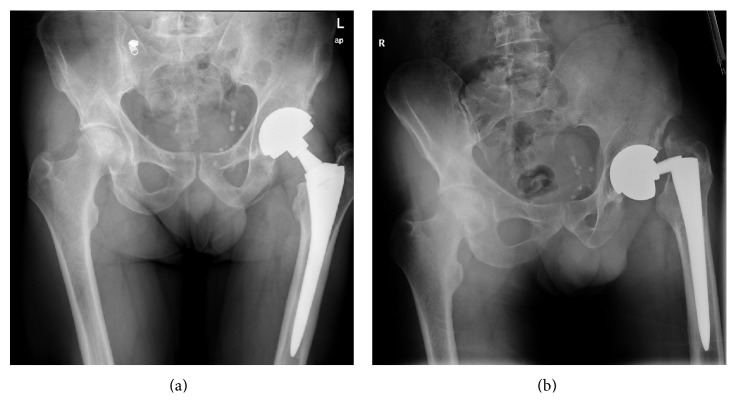
(a) Plains radiographs of the pelvis showing a Profemur Modular stem and Conserve Cup (Wright Medical Technology, Inc., 5677 Airline Road, Arlington, TN 38002) with a long modular titanium neck with 8 degrees of varus, metal on metal bearing surface, and a 50 mm big ball. (b) Emergency radiographs of the pelvis showing a rupture of the modular neck.

The present case was a 53-year-old male with a BMI of 28, type 2 diabetes, dyslipidemia, and a nonfunctional hypophyseal adenoma that was surgically removed in 1994. Due to the state of panhypopituitarism, the patient developed an avascular necrosis of both femoral heads, primarily of the left hip. A total hip arthroplasty of the left hip was performed in 2009 using a Profemur Modular stem and Conserve Cup (Wright Medical Technology, Inc., 5677 Airline Road, Arlington, TN 38002) with a long modular titanium neck with 8 degrees of varus, metal on metal bearing surface, and a 50 mm femoral head. Postoperative radiographic control showed a correctly placed prosthesis conserving patient's natural offset and varus reproducing the native hip's biomechanics (Figure 1(a)).

**Figure 2:**
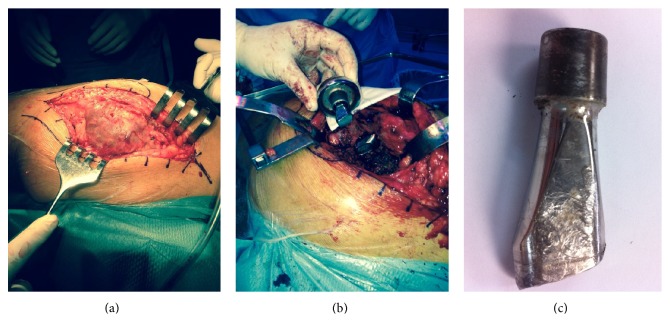
(a) An important pseudotumoral well-organized periprosthetic tissue reaction was found surrounding the joint. (b, c) The rupture of the modular neck was confirmed; failure of the implant seemed to have originated on the anterior aspect of the tapper junction.

**Figure 3:**
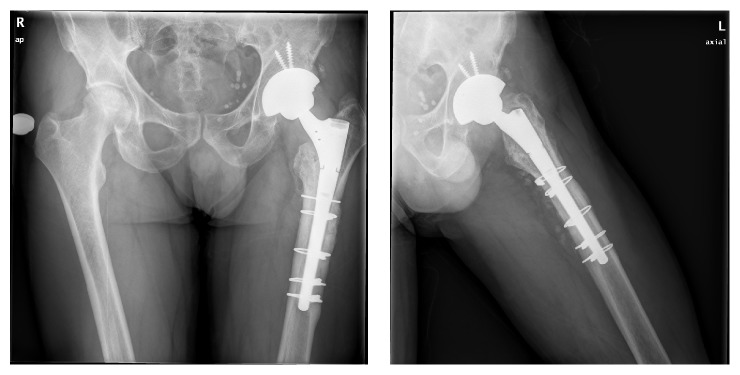
Plain radiographs of the pelvis showing a straight Revitan revision prosthesis (Zimmer, Warsaw, IN) and a TMT revision cup (Zimmer, Warsaw, IN) with press fit and two cancellous screws; the trochanteric osteotomy was synthetized with 4 cerclage wires.

Follow-up controls at three, six, and twelve months showed no radiological findings nor are any complaints from the patient documented. Three and a half years after the primary THA, the patient attended the emergency department due to acute left hip pain with no particular traumatism or abrupt movements. Radiological examination confirmed a fracture of the modular neck (Figure 1(b)). A revision surgery was performed; through a posterolateral approach a considerable pseudotumoral well-organized periprosthetic tissue reaction was found surrounding the joint; debridement was performed (Figure 2(a)). The pseudotumor did not infiltrate nor destroy any skeletal or muscular structures. The rupture of the modular neck was confirmed (Figure 2(b)). Failure of the implant seemed to have originated on the anterior aspect of the taper junction (Figure 2(c)). An extended trochanteric osteotomy was performed since the extraction of the remainder modular neck did not seem feasible. A straight Revitan revision prosthesis (Zimmer, Warsaw, IN) was placed and the osteotomy synthetized with 4 cerclage wires. Acetabular component was revised using a TMT revision cup (Zimmer, Warsaw, IN) with press fit and two cancellous screws (Figure 3).

## 3. Discussion

Correctly reproducing offset, anteversion, and length is crucial for soft tissue balance, decreasing impingement, and restoring the hip's rotational center [7]. Due to such potential advantages, modularity has been increasing in popularity in the last two decades [1, 2, 11]. Nevertheless, recent concerns with modularity have arisen due to the idea that the increasing number of interphases may very well increase such complications as fretting corrosion [1, 13], metal on metal debris, and fatigue failure of the modular junctions [5, 12]. Some authors report no increase in corrosion or metal ion release at the junctions in simulated* in vivo* conditions [14] whereas other* in vitro* experimental models have reported an increase in debris/corrosion at the modularity junctions leading to osteolysis and more susceptibility of fatigue fracture [13, 15].

A recent study published by Duwelius et al. [10] reports no differences between modular and nonmodular THAs. It consists of a retrospective observational study with almost 900 patients divided into two different cohorts (284 nonmodular THAs; 594 modular THAs). No significant differences were found between modular or nonmodular prosthesis when evaluating clinical hip scores, complications, or the need for revision surgery after a 2.4-year mean follow-up. In this particular study, no failure of the modular taper junctions is described.

Evaluating the present case, the significant soft tissue and pseudotumoral periprosthetic tissue reaction that was observed intraoperatively suggest that a reasonable amount of corrosion and metal fatigue were present and would probably explain the ultimate failure of the modular neck. Nonetheless, metal on metal bearing surface and big femoral heads have been related to metal ion release and may contribute to the metal debris soft tissue reaction in this case. Corrosion at the Morse taper, once the head was disassembled, suggests that this was indeed the case. Combination of a big femoral head and a metal on metal bearing has been involved in metal ion release of an corrosion, not only because of the metal on metal pairing, but also because of the increased strain force at the taper caused by the oversized femoral head [5, 6]. In the present case, a metal cup was chosen in a time when studies comparing different metal alloys and their interactions were beginning to be published. Such studies suggest that fretting and crevice corrosion are real concerns for both titanium and cobalt-chrome (Co-Cr) alloys in a clinical setting [11, 16, 17].* In vitro*, titanium modular necks have shown 38% less load bearing capacity when compared to Co-Cr, which also showed 1000 times longer fatigue life than titanium [15]. The considerable metal debris reaction and corrosion described in the present case seem to originate from a combination of head size, bearing surface, and titanium alloy at the taper junctions.

A handful of case reports have been published regarding modular neck fractures in THA and the patients seem to have some similarities between them, which the patient in the current case also seems to share [11, 12, 18]. Subjects are described as middle-aged, overweight, or obese patients usually with an active lifestyle and bearers of a long varus modular neck. Skendzel et al. reported two cases of modular neck failure and emphasized that the patients' obesity and, in particular, the use of a long varus neck may play a decisive role in such implant failures since the bending moment of a long varus neck is increased in over 30% when compared to a standard short neck [18]. The present case combines a series of factors (overweight active patient, modular titanium long varus neck, oversized femoral head, and metal on metal bearing), which may constitute potential sources of fretting corrosion and failure of modular implants which should be taken into account in preoperative planning.

Various circumstances such as large femoral head with a long varus neck, corrosion, patients' BMI, and activity level may participate in creating the necessary environment for fatigue failure of a modular hip replacement implant. In our case, the patient presented double risk for implant failure due to the long varus neck and big femoral MoM bearing. Modularity may have its indications since it serves as a very interesting tool for reproducing natural hip biomechanics; nonetheless, patients and modular configuration should be carefully selected.
